# New Appliances and Things Medical

**Published:** 1904-08-13

**Authors:** 


### NEW APPLIANCES AND THINGS MEDICAL.

### CHAPMAN'S FOOD.

(Chapman's Food Company, Battersea, London.)

This food consists essentially of Chapman's whole wheat flour. To make the food the whole wheat flour is roasted, and by this means some of the original starch is converted into dextrin and some into glucose, or in other words, into more soluble carbohydrates. The food thus compares very favourably with the whole wheat flour itself. It may, perhaps, be mentioned that whole wheat flour contains more nitrogenous matter and more phosphates than ordinary wheat flour, and in addition a substance called cerealin, which is thought to be capable of partially digesting starch, converting it into dextrin. The flavour of Chapman's food is pleasant, and, in view of its composition, it seems to us a useful preparation.

